# Custodian Controlled Data Repository - supporting the timely, easy and cost effective access to linked data

**DOI:** 10.23889/ijpds.v1i1.346

**Published:** 2017-04-19

**Authors:** Christopher Radbone, Suzi Adams

**Affiliations:** 1 SA NT DataLink

## Objectives

In response to Data Custodian and Researcher's request to assist improving the timeliness and ease of data extractions, SA NT DataLink established the Custodian Controlled Data Repository (CCDR). Issues including conflicting work priorities and limited resources do prevent timely data extractions. SA NT DataLink, as a trusted third party enable Custodians to hold separate copies of their de-identified content data ready for timely release. The CCDR is opt-in for Custodians. Its key strength being the data remains under the control of participating Custodians, with Custodians updating and correcting their data. SA NT DataLink staff are work with Data Custodians to clean, standardise, update and prepare data in advance of anticipated approvals, including spatial enabled GIS variables. SA NT DataLink staff work under the direction of the Data Custodians, taking responsibility for preparing the data extractions required for the approved project.

## Approach

The SA NT DataLink Custodian Controlled Data Repository (CCDR) takes advantage of the Secure Unified Research Environment (SURE), which is a secure remote access data laboratory operated by the Sax Institute in Sydney Australia. Using thin client and two-factor authentication, Data Custodians from South Australia and across Australia are able to securely store and maintain de-identified copies of their content data in SURE, ready for standardisation, quality review, and more timely release for approved use and data linkage projects.

## Results

The CCDR functional diagram provides an understanding of the data flow and processes that support more timely, easy and cost effective collation and provision of de-identified and privacy protected data. The Curated Gateway feature of SURE manages all the data coming into and being released from the Repository. Agreed regular updates of Data Custodian's data is able to be stored into their sub-directory, with access to the sub-directory managed by authentication and passwords. The SA NT DataLink Analysts is able to perform the role of data integrator for approved projects and use, also running privacy protecting algorithms and verification of the data being provided against the approvals. 

## Conclusion

The CCDR securely stores the de-identified data ready for it to be integrated and released to Researchers. Use of secure remote access technologies allows Data Custodians to maintain control of their preloaded and pre-cleaned data in CCDR. In doing so this allows Custodians to authorise the use, from which SA NT DataLink staff dedicated to working only in the CCDR, integrate and release the data in a more timely manner.

